# Early-Onset Neonatal Pneumococcal Sepsis: An Old but Sometimes Forgotten Pathogen

**DOI:** 10.7759/cureus.29403

**Published:** 2022-09-21

**Authors:** Inês Coelho, Sofia Baptista, Ana Raquel Ramalho, Claúdia Calado, Luísa Gaspar, Maria João Virtuoso, João Rosa

**Affiliations:** 1 Pediatrics Department, Centro Hospitalar Universitário do Algarve, Faro, PRT; 2 Neonatal Intensive Care Unit, Centro Hospitalar Universitário do Algarve, Faro, PRT

**Keywords:** neonatal emergency, vaginal colonization, streptococcus pneumoniae, neonatal care, neonatal sepsis

## Abstract

*Streptococcus pneumoniae* (SP) is an uncommon but potentially serious neonatal pathogen. SP is perceived as a significant cause of mortality and morbidity in infancy; however, there are relatively few cases of neonatal sepsis recorded, with an incidence between 1% and 11%. We aim to report the spectrum of morbidity associated with SP infections in the neonatal period.

Two cases of neonatal SP infection are reported. The first neonate presented with a very early onset of severe clinical disease with bacteremia and pneumonia. She developed severe pulmonary hypertension and needed intensive ventilatory support, including nitric oxide, and vasoactive drugs. An SP serotype 23B was isolated from blood cultures and bronchial secretions as well as from the mother’s vaginal secretions. In the second case, the baby presented with bacteremia and meningitis. He remained hemodynamically stable and did not need respiratory support. Blood and cerebrospinal fluid cultures revealed an SP serotype 8. In both cases, the neonates were treated with vancomycin and cefotaxime. Both mothers remained well and asymptomatic during the perinatal period.

These reported cases emphasize the importance of considering a wide range of microorganisms in the differential diagnosis of early-onset neonatal sepsis. Although uncommon, SP can have different clinical manifestations and cause significant diseases in newborns. Specific preventive measures against early-onset sepsis for this pathogen are yet to be implemented due to the absence of sufficient scientific evidence. For this reason, prompt and aggressive treatment remains the best therapeutic approach.

## Introduction

*Streptococcus pneumoniae *(SP) infection is one of the most frequent causes of death in developed countries in the general population [1]. However, it is an uncommon but potentially serious neonatal pathogen. Recorded cases of neonatal sepsis are relatively few, with a described incidence between 1% and 11% [2]. The clinical manifestations during the neonatal period do not differ from those presented by other bacteria such as *Streptococcus agalactiae *and *Escherichia coli*, but *S. pneumoniae *presents higher morbidity and mortality, described as up to 50% [2-4].

Given its high morbidity and mortality, it should be considered an agent responsible for neonatal sepsis, mostly in those of early onset. We aim to report the spectrum of morbidity associated with SP infections in the neonatal period.

## Case presentation

Case 1

A female infant was born by normal vaginal delivery at 38 weeks to a primigravida healthy mother. Spontaneous rupture of membranes occurred 28 hours before delivery. The mother was in good health condition during the perinatal period. The baby was born in good condition, weighing 3,110 g, and stayed with her mother in the perinatal ward.

The baby developed respiratory distress at around 12 hours of life and was therefore admitted to the neonatal intensive care unit (NICU). For worsening work of breathing, the neonate required tracheal intubation at 20 hours of life. She developed severe pulmonary hypertension over the next few hours and needed intensive ventilatory support including nitric oxide and inotropic support. Chest radiographs were consistent with bilateral pneumonia.

Ampicillin (200 mg/kg/day) and gentamicin (4 mg/kg/day) were started as empirical antibiotic therapy. There was no clinical improvement, and on day 3, antibiotics were changed to cefotaxime and vancomycin. In terms of laboratory findings, she had an elevated C-reactive protein (CRP) (maximum of 55 mg/L on day 4), leucopenia (2.9 × 10^9^/L), and persistent thrombocytopenia (lower of 10,000/mL on day 4) with five platelet transfusions (Table 1).

**Table 1:** Blood tests

Tests	Values
Leukocytes	2.9 × 10^9^/L
Platelets	Lower: 10,000/mL (day 4)
C-reactive protein	Maximum: 55 mg/L (day 4)

We were unable to perform a lumbar puncture due to her critical clinical condition. The blood culture and the bronchial secretions obtained on the first day of life grew SP, sensitive to ampicillin and cefotaxime. Serotyping was performed, which revealed an SP serotype 23B (Table 2).

**Table 2:** Newborn microbiological cultures

Microbiological cultures	
Blood culture	SP serotype 23B
Bronchial secretions	

Due to the severity of the baby’s clinical condition and to the fact that she only improved after the antibiotic change, a decision was made to continue treatment with cefotaxime and vancomycin for seven and 21 days, respectively. The mother’s vaginal swab revealed the same SP serotype. The mother had none of the vaccines against SP and remained asymptomatic throughout the perinatal period.

Case 2

A male infant was born at 38 weeks gestation, the first child of healthy young parents. The pregnancy had a poor follow-up, and the rectovaginal swab was not performed. There was a spontaneous rupture of membranes five hours prior to delivery with clear amniotic fluid. The infant was born in good condition, with an Apgar score of 9/10 and weighing 2,745 g.

Due to persistent grunting, hypotonia, and poor peripheral perfusion, he was admitted to the NICU at around 20 hours of life. Laboratory evaluation showed leukocytes of 3,500/mm, with 60% neutrophils, and CRP of 42 mg/L (Table 3). Lumbar puncture was performed, with a yellow citrus cerebrospinal fluid and no cytochemical abnormal findings (Table 3). Ampicillin (200 mg/kg/day) and gentamicin (4 mg/kg/day) were started as empirical antibiotic therapy.

**Table 3:** Auxiliary diagnostic tests

Tests	Results
Lumbar puncture	No cytochemical abnormal findings
Leukocytes/C-reactive protein	3.5 × 10^9^/L (60% neutrophils)/maximum: 109 mg/L (day 3)
Microbiological cultures (blood and cerebrospinal fluid)	SP serotype 8

Both blood and cerebrospinal fluid cultures grew SP, and antibiotic therapy was changed to vancomycin and cefotaxime. Serotyping was performed, which revealed an SP serotype 8 (Table 3). The antibiotic sensitivity profile showed susceptibility to penicillin, cefotaxime, and vancomycin, as well as to all other tested antibiotics. Vancomycin was stopped, and cefotaxime was maintained for 21 days.

The baby remained hemodynamically stable and did not require respiratory support. His highest recorded CRP was 109 mg/L on day 3. Imaging studies confirmed the presence in the thymus and spleen. Transfontanellar ultrasound was unremarkable. The mother remained asymptomatic and also did not have any of the vaccines against SP. A culture of vaginal secretions was not obtained.

## Discussion

Neonatal SP infections are rare, with sporadic cases and small series of sepsis, pneumonia, and meningitis reported in the literature [1]. It is estimated to account for 1%-11% of all cases of sepsis in infants [1,2].

Hoffman et al. [2] reported a total of 29 cases of SP infection in neonates, eight corresponding to acute otitis media and 21 to invasive bacterial infections (meningitis, 27%; bacteremia, 28%; pneumonia, 14%; osteoarticular infections, 3.4%). Only three patients presented with early systemic infection (within the first 72 hours) and only one before 24 hours of life. The average age of presentation was 18 days, and 90% were term neonates. Of the babies with invasive infection, 30% had leukopenia (white blood cells < 5,000/mm^3^). The most frequent serogroup was 19, both in invasive infections (30%) and in acute otitis media (32%). Of the isolates, 20% were resistant to penicillin, and one was classified as non-susceptible to third-generation cephalosporins. Mortality in this series was 14.3%, very different from what is described for children over two months of age (1.3%) where fatal cases occur mainly in children with underlying diseases [2]. All deaths occurred in the first 36 hours of disease [2].

In both our cases, symptoms started in the first 24 hours of life. In the series of Hoffman et al., that was the case in only one out of 29 neonates [2]. For the first neonate reported in this article, there was a severe clinical deterioration with intensive ventilatory and vasoactive support needed, while the second baby remained hemodynamically stable and had no need for respiratory support. For both our cases, the SP was sensitive to penicillin and cefotaxime; Hoffman et al. described a 20% rate of resistance to penicillin [2].

The risk factors related to infection by SP in newborns are premature rupture of membranes, prematurity, neonatal pneumonia with early presentation (<72 hours), and vaginal delivery (the risk factor most frequently associated) [4]. For this reason, despite the low frequency of SP as a cause of neonatal illness, it is important to consider this microorganism among the agents of sepsis in newborns.

Regarding the pathogenesis of invasive infection of the newborn by SP, the major role has been attributed to vertical transmission associated with maternal vaginal carriage and/or endometritis [2-7]. Through molecular biology studies, the clonal transmission of the bacteria from mother to child has been verified [5,8].

In the cases presented in our article, the origin of the infection was assumed to be a vertical transmission, and this hypothesis was confirmed in the first case with isolation of the same SP serotype in a culture of the mother’s vaginal swab. The frequency of vaginal colonization by SP is very low (0.03%-0.75%) [2,9]. Studies published in the past decade warned about a possible increase in vaginal colonization by this agent, due to changes in sexual practices during pregnancy (e.g., oro-genital sex) and the development of better diagnostic techniques [5,8].

An additional concern is the possible vaginal colonization of mothers with strains of SP resistant to penicillin and third-generation cephalosporins or the selection of resistant strains secondary to the use of antimicrobials for the prevention of neonatal *S. agalactiae* infection [5]. In the international scientific literature, it has been described that between 20% and 50% of *S. pneumoniae* strains isolated from newborns would be resistant to penicillin and that between 4% and 7% would be resistant to third-generation cephalosporins [2,7,8].

Therefore, some authors, namely, McDonald et al. [5], argue that if the incidence of neonatal sepsis due to *S. pneumoniae *increases to the point where specific prevention measures were required, maternal vaccination with pneumococcal vaccine 13-valent or 23-valent in the third trimester could be considered a useful practice [3,5,10-12].

Prevenar 13®, the 13-valent pneumococcal polysaccharide conjugate vaccine, is included in the Portuguese national vaccination program. However, it does not contain all of the *Streptococcus pneumoniae *serotypes considered the cause of early-onset sepsis 1 to 12, 14, 17, 18, 19, 23, 27, 28, 31, and 39 [3,10-13]. In our report, the serotypes were 23B and 8, neither of them is included in Prevenar 13®, but the last one is included in Pneumovax 23®.

On the other hand, a recent Cochrane library review on the subject has found that there was no evidence of neonatal infection prevention as an effect of pneumococcal vaccination during gestation, although it was based on only a few studies, with overall poor quality of evidence [14].

Other authors defend that another possible prevention strategy would be testing for SP in vaginal swabs and starting antibiotic prophylaxis during labor when positive [3,10-13]. However, due to the low incidence of neonatal sepsis caused by SP and the low prevalence of maternal vaginal colonization (0.03%-0.75%), the systematic screening is probably not cost-effective; nevertheless, it should be considered in pregnant women with a history of a previous neonate with invasive disease [3,9].

There is not yet sufficient scientific evidence to support the implementation of these measures. In addition to the evaluation of these and other possible prevention strategies, supplementary studies on epidemiologic surveillance and the determination of risk factors related to neonatal *Streptococcus pneumoniae* sepsis are essential.

## Conclusions

These reported cases emphasize the importance of considering a broad spectrum of microorganisms in the differential diagnosis of early-onset neonatal sepsis, and while uncommon, SP can have different clinical manifestations and cause significant disease in newborns. Specific preventive measures against early-onset sepsis for this pathogen are yet to be implemented due to the absence of sufficient scientific evidence. For this reason, prompt and aggressive treatment remains the best therapeutic approach.
